# Single molecule force spectroscopy data and BD- and MD simulations on the blood protein von Willebrand factor

**DOI:** 10.1016/j.dib.2016.07.031

**Published:** 2016-07-21

**Authors:** Sandra Posch, Camilo Aponte-Santamaría, Richard Schwarzl, Andreas Karner, Matthias Radtke, Frauke Gräter, Tobias Obser, Gesa König, Maria A. Brehm, Hermann J. Gruber, Roland R. Netz, Carsten Baldauf, Reinhard Schneppenheim, Robert Tampé, Peter Hinterdorfer

**Affiliations:** a Department of Applied Experimental Biophysics, Institute of Biophysics, Johannes Kepler University, Linz, Austria; b Molecular Biomechanics Group, Heidelberg Institute for Theoretical Studies, Heidelberg, Germany; c Department of Physics, FU Berlin, Berlin, Germany; d Center for Advanced Bioanalysis GmbH (CBL), Linz, Austria; e Department of Pediatric Hematology and Oncology, University Medical Center Hamburg -Eppendorf, Hamburg, Germany; f Theory Department, Fritz-Haber-Institut der Max-Planck-Gesellschaft, Berlin, Germany; g Institute of Biochemistry, Biocenter, Goethe-University Frankfurt, Frankfurt/Main, Germany

**Keywords:** Atomic force microscopy, Single molecule force spectroscopy, Molecular dynamics simulation, Brownian dynamics simulation, von Willebrand factor

## Abstract

We here give information for a deeper understanding of single molecule force spectroscopy (SMFS) data through the example of the blood protein von Willebrand factor (VWF). It is also shown, how fitting of rupture forces versus loading rate profiles in the molecular dynamics (MD) loading-rate range can be used to demonstrate the qualitative agreement between SMFS and MD simulations. The recently developed model by Bullerjahn, Sturm, and Kroy (BSK) was used for this demonstration. Further, Brownian dynamics (BD) simulations, which can be utilized to estimate the lifetimes of intramolecular VWF interactions under physiological shear, are described. For interpretation and discussion of the methods and data presented here, we would like to directly point the reader to the related research paper, “*Mutual A domain interactions in the force sensing protein von Willebrand Factor*” (Posch et al., 2016) [1].

**Specifications Table**

**Table:** 

Subject area	*Biophysics*
More specific subject area	*Single molecule force spectroscopy (SMFS), Brownian dynamics simulations (BD), Molecular dynamic (MD) simulations*
Type of data	*Graph, figure, equations*
How data was acquired	*Pico SPM Plus setup (Agilent Technologies, Chandler, AZ, USA): atomic force microscopy, single molecule force spectroscopy*
	*Molecular dynamics simulation*
	*Brownian dynamics simulation*
Data format	*Analyzed*
Experimental factors	*AFM cantilever tips as well as mica supports were chemically functionalized with different VWF constructs*[1]
Experimental features	*This data in brief article describes how different methods, such as AFM based single molecule force spectroscopy and BD- and MD simulations can be used to study the protein VWF.*
Data source location	*Protein purification: Hamburg, Germany, Latitude: 53.55, Longitude: 10, GPS: 53° 33*׳ *0*" *N, 10° 0*׳ *0*" *E*
	*Single molecule force spectroscopy: Linz, Austria, Latitude: 48.3, Longitude: 14.3, GPS: 48° 18*׳ *0*" *N, 14° 18*׳ *0*" *E*
	*MD simulations: Heidelberg, Germany, Latitude: 49.4166667, Longitude: 8.7, GPS: 49° 25*׳ *0*" *N, 8° 42*׳ *0*" *E*
	*BD simulations: Berlin, Germany, Latitude: 52.5166667, Longitude: 13.4, GPS: 52° 31*׳ *0*" *N, 13° 24*׳ *0*" *E*
Data accessibility	*All data is with this article*

**Value of the data**

• Single molecule force spectroscopy (SMFS), which is described in this article, is a technique to nondestructively probe the forces and the dynamics between molecules under physiological conditions. In combination with our chemical expertize, this powerful tool is applicable for almost every receptor/ligand combination, which offers many possibilities for new collaborations.
• The detailed description of the calculation of tensile force profiles along a protein chain from BD simulations might be a guideline for other researcher pursuing similar interests.
• The fitting of rupture forces versus loading rate profiles in the MD loading-rate range show an elegant way for the comparison of data from experimental AFM experiments and theoretical MD simulations and provide for collaboration between experimentalists and theorists.

## 1 Data

**Fig. 1:**
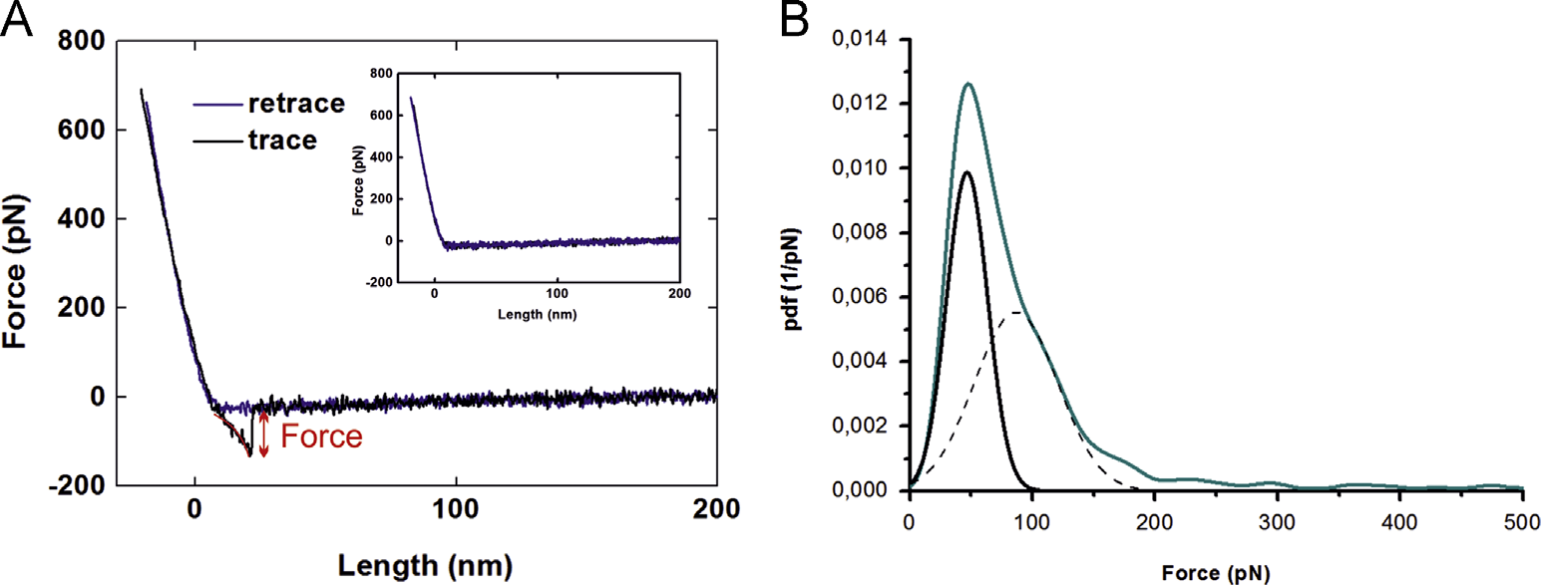
(A) Example of a typical force distance cycle (FDC) showing a specific VWF A1/A2 unbinding event, which was marked with a polynomial fit. The unbinding force was determined from the jump at the point of dissociation as indicated. In the blocking experiment (Fig. 2) no specific unbinding events occurred (Insert in A). At least 1000 FDCs were recorded for each loading rate to create a typical force distribution as shown in (B). The distribution was taken from the A1/A2 interaction measurement for a velocity of 400 nm/s. A Gaussian distribution was fitted to the first peak and forces within the interval *µ*±*σ* were used for further analysis in the loading rate dependence plot (Fig. 3). The loading rate r was calculated by multiplying the pulling velocity v with the effective spring *k*_eff_ (slope at the rupture) of the system.

**Fig. 2:**
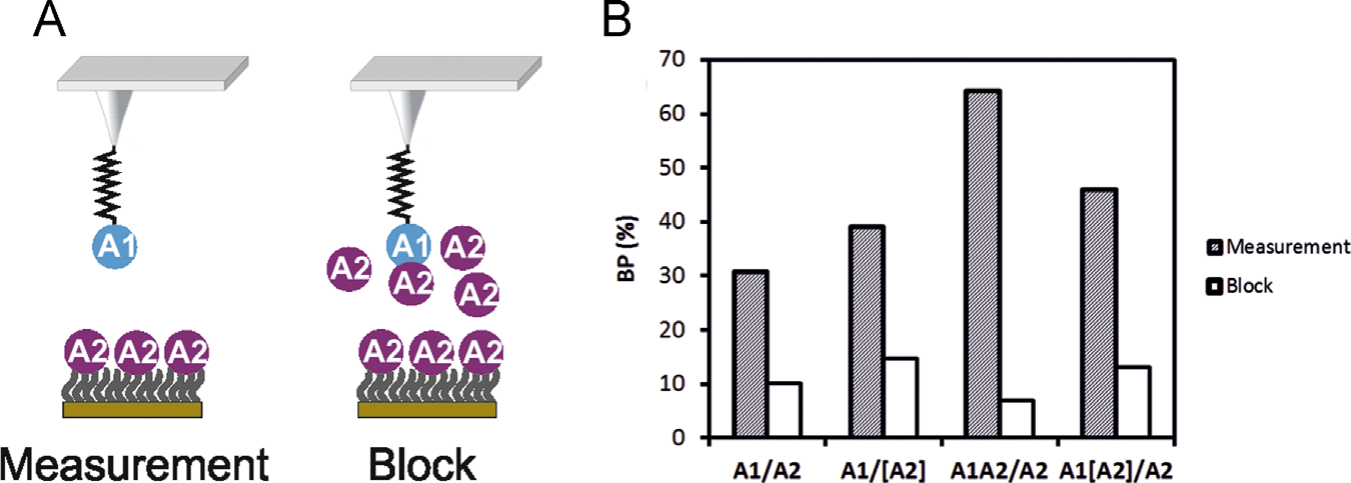
Specifity proof measurement of the VWF A1/A2 interaction studies. After the actual measurement, free VWF A2 domain constructs (*c*=0.1 mg/ml, 2 h) were injected into the measuring buffer. The domain on the tip was blocked and thus incapable of binding to the A2 domains on the sample surface. As a consequence, the binding probability (BP) significantly decreased and thus proved the specifity of the interaction. (Subset on the data shown in Fig. 1[1]).

**Fig. 3:**
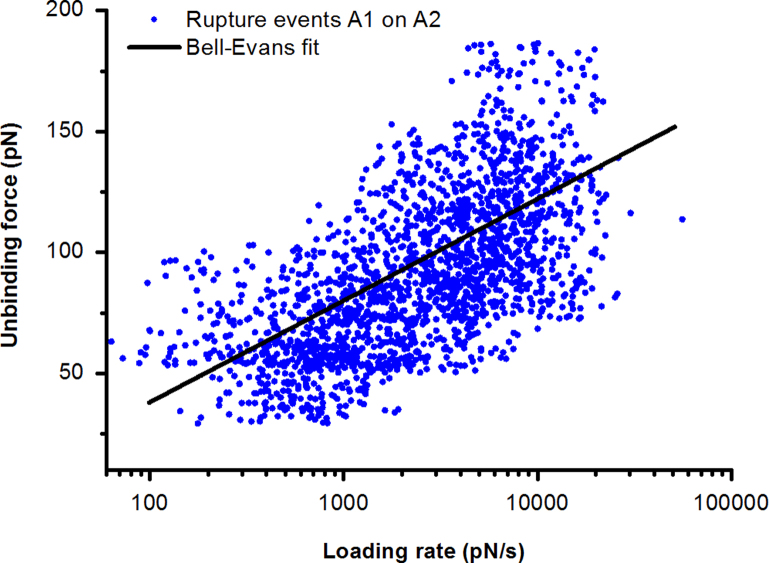
Loading rate dependence plot (LRD) for the A1/A2 measurement. The data points, representing a single unbinding event each, were fitted with a single energy barrier binding model using a maximum likelihood fitting routine to obtain the kinetic off-rate *k*_off_.

**Fig. 4:**
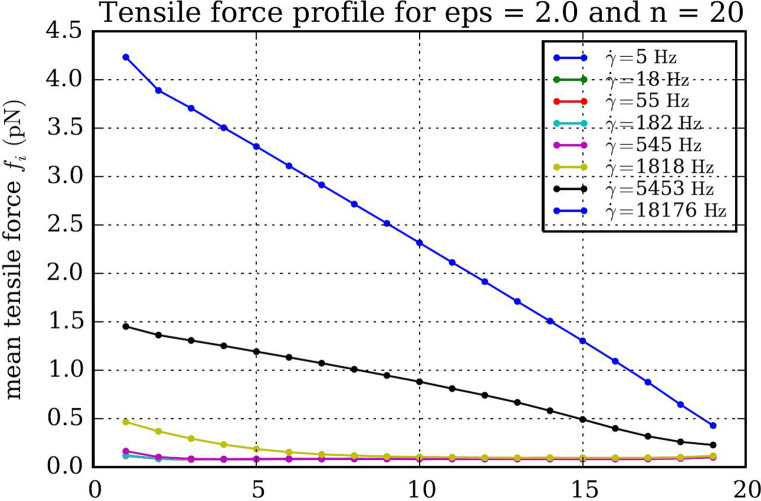
Tensile force profile for eight different shear flows (5.5, 18, 55, 182, 545, 1818, 5453, 18,175 s^−1^) and one contour length of 1.14 μm. Tensile force profiles for 182, 55, 18 and 5 Hz overlap almost entirely.

**Fig. 5:**
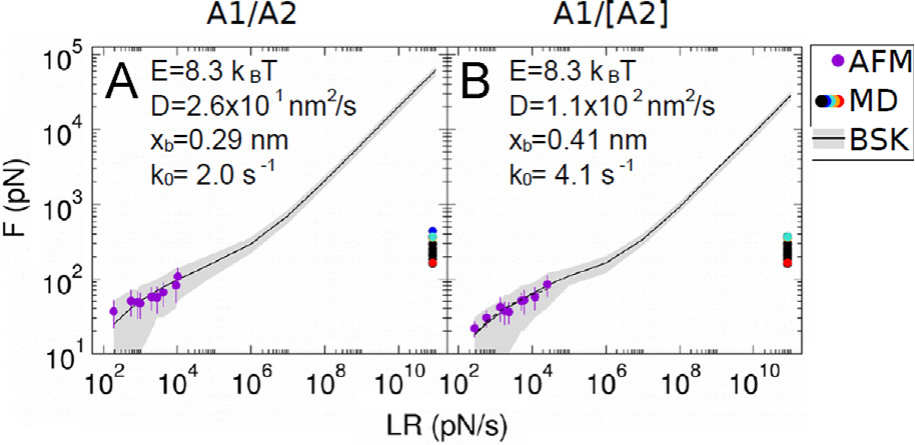
Unconstrained rupture forces of the A1-A2 complex as a function of the applied loading rate, for the A1-A2 wild-type complex A1/A2 (A) and for its bridged mutant A1/[A2] (B). Forces measured by AFM (average± stdev) and computed from MD simulations are shown with dots. For the A1/[A2] bridged construct, the largest MD rupture force, observed upon unfolding of the A2 domain, was excluded. Forces were fitted using the Bullerjahn, Sturm and Kroy (BSK) model (Bullerjahn et al. 2014). Fitting was carried out using exclusively the AFM data. Average (solid line) and the 95% confidence interval (gray area) are shown. (*E*, *D*, *x*_b_, *k*_0_) fitting parameters are indicated in each panel.

In Fig. 1 we provide deeper understanding of the recording of force distance cycles and advanced data evaluation. The process of specifity proof measurements using the example of VWF A1/A2 interaction studies is discussed and a typical loading rate dependence plot is shown in Fig. 2, Fig. 3. The BD simulation part ((1), (2), (3), (4), (5), (6) and Fig. 4) gives a detailed description of the calculation of force profiles for protein chains and the fitting of rupture forces versus loading rate profiles in the MD loading-rate range can be found in Fig. 5.

## 2 Experimental design, materials and methods

### 2.1 AFM

#### 2.1.1 Experimental design

SMFS measurements were performed using a Pico SPM Plus setup (Agilent Technologies, Chandler, AZ, USA) under physiological conditions. Single VWF A-domains or VWF A domain constructs were either coupled to the AFM tip or to the sample surface. For SMFS experiments non-conductive Silicon Nitride MSCT tips (Brucker Corporation, MA, USA) with small spring constants (*k*=0.03 N/m) were utilized. The actual spring constant was determined using the thermal noise method [2]. AFM cantilever tips as well as mica supports were chemically functionalized with different VWF constructs [1].

#### 2.1.2 Materials

All chemicals were used in the highest available purity. 3-Aminopropyl-triethoxy silane (APTES; Sigma Aldrich, Vienna, Austria) was distilled at low pressure and stored under argon in sealed crimp vials over dry silica gel (to avoid polymerization) at −20 °C. MilliQ (Millipore, Massachusetts, USA) purified water was used for all aqueous solutions. Triethylamine (TEA, Sigma Aldrich, Vienna, Austria) was stored under argon and in the dark to avoid amine oxidation. Chloroform was purchased from J.T. Baker (Griesheim, Germany), Argon and N_2_ from Linde Gas GmbH (Stadl-Paura, Austria). Concentrated HCl was purchased from Sigma Aldrich (Vienna, Austria). Ethylenediaminetetraacetic acid (EDTA) and Tris base were purchased from VWR International (Vienna, Austria), Hepes and NiCl_2_ were obtained from Merck (Darmstadt Germany) and TCEP (tris(2-carboxyethyl)phosphine) hydrochloride from Invitrogen (Vienna, Austria). Disulfide-tris-NTA was generously provided by the Tampé lab, Biocenter, Frankfurt am Main, Germany. The heterobifunctional crosslinker maleimide-PEG_27_-NHS was purchased from Polypure (Oslo, Norway). N-succinimidyl 4-(dimethoxymethyl)benzoate (SDMB) was synthesized as described in [3]. The cDNAs’ coding for recombinant human VWF constructs containing the A1A2 (aa 1230-1672) construct, the single A1 (aa 1230-1463) and the single A2 (aa 1494-1672) were cloned into the mammalian expression vector pIRES neo2 [4]. All VWF constructs were labeled with a His_6_-tag. Mutations were inserted by site-directed mutagenesis employing the QuickChange kit (Stratagene). The vectors were used to transform Top10 super competent cells (Invitrogen) and sequenced. Plasmid purification was performed using the Endofree Plasmid Maxi Kit (QIAGEN). HEK293 cells were cultured in Dulbecco Modified Eagle Medium (DMEM, Invitrogen) with 10% [v/v] fetal bovine serum (Invitrogen) and 1% penicillin/streptavidin at 37 °C and 5% CO_2_. These cells were transfected with the VWF vectors using Lipofectamine 2000 (Invitrogen) according to the manufacturer׳s instructions and recombinant expression of VWF variants was performed as previously described [5]. His-tagged VWF domain constructs were purified employing the His-Pur Ni-NTA Resin (Thermo Fisher Scientific) according to the manufacturer׳s instruction for purification of His-tagged proteins using a gravity-flow column. Mica sheets were bought from Christine Groepl, Electron Microscopy (Tulln, Austria). For aqueous solutions, TBS buffer (50 mM Tris, 150 mM NaCl, pH 7.4) and Hepes buffer (prepared from a 1 M solution of Hepes acid by adjustment of pH 7.5 or pH 9.6 – as stated in the text – with 20% NaOH) were used.

#### 2.1.3 Methods

Interactions were probed by conducting force-distance-cycles (FDCs) at different loading rates r, i.e., at nine different velocities (50, 100, 200, 400, 600, 800, 1200, 2000 and 3000 nm/s). To gain reliable statistics, at least 1000 FDC were recorded at each pulling speed. The position of the tip relative to the surface was changed every 200 FDC, so as to statistically avoid position dependent artifacts. Unbinding events in the recorded FDC were marked with a polynomial fit (Fig. 1(A)). Binding events could be discerned from nonspecific adhesion by a characteristic parabolic force signal due to the elastic properties of the linkers. To additionally prove the specificity of the interactions, blocking experiments were performed. In this process, the ligand on the tip (e.g. VWF domain A1) was incubated with free receptors (e.g. VWF domains A2) in solution for two hours (Fig. 2). The free receptors saturate the ligand on the tip, and thus block the interaction between the ligand and the receptor on the sample surface. Thereafter, almost no specific interactions were observed in the FDC and the binding probability (BP), defined as the ratio between FDCs showing an unbinding event relative to the total number of FDCs, dramatically decreased (Fig. 2).

Probability density functions (pdf) of the measured forces were computed for each loading rate. A Gaussian distribution was fitted to the first peak of the pdf and forces within the interval *µ*±*σ* were used for further analysis (Fig. 1(B)). The unbinding forces were plotted against the logarithm of the loading rate r, which is given by the product of the effective spring constant *k*_eff_ (slope at the rupture of an unbinding event, see Fig. 1(A)) and the pulling velocity *v*. The data points in this loading rate dependence plot (LRD) represent a single unbinding event each and were fitted with a single energy barrier binding model [6], using the maximum likelihood fitting routine [7] to obtain the kinetic off-rate k_off_ and the width of the energy barrier *x*_β_. A typical example for such a data cloud and its fit is shown in Fig. 3.

### 2.2 Calculating tensile force profiles (under shear flow conditions) from BD simulations

We used Brownian dynamics including hydrodynamic interactions [8] to simulate the behavior of VWF under shear flow conditions. The mean tensile force along the backbone at the grafted chain end was then plugged into the Bell Evans Eq. (1), see also [1]:

(1) $$\tau \left(F\right)=\tau_{0}e^{\frac{-x_{\beta }F}{k_{B}T}},$$

to gain an estimate for the lifetime of the A1/A2 complex under physiological conditions. In the following, we will expand on the model and the parameter values we used and how we calculated the tensile force profiles from the simulation.

#### 2.2.1 The model

A linear chain of VWF protomers is modeled as N beads interacting by a Lennard-Jones potential. The backbone of the chain is additionally held together by a spring interaction. The potential energy of the chain therefore reads

(2) $$U_{\mathrm{bead}-\mathrm{spring}}=\varepsilon \overset{N}{\underset{i<j}{\sum }}\left[{\left(\frac{2a}{r_{\mathrm{ij}}}\right)}^{12}-2{\left(\frac{2a}{r_{\mathrm{ij}}}\right)}^{6}\right]+\frac{\kappa }{2}\sum_{i=1}^{N-1}{\left(r_{i,i+1}-2a\right)}^{2}$$

where $r_{i}$ is the position vector of bead *i* and $r_{\mathrm{ij}}$ is defined by the norm of the distance vector $r_{i}-r_{j}$. The parameters ε,a and κ are the cohesion strength, the bead radius and the spring constant, respectively. The repulsive potential of the surface was chosen to be:

(3) $$U_{\mathrm{surface}}=\sum_{i=1}^{N}\{\begin{array}{c} \frac{2\pi {k_{B}T\sigma }_{R}}{a}\left(\frac{2}{5}{\left(\frac{\sigma_{R}}{z_{i}}\right)}^{10}-{\left(\frac{\sigma_{R}}{z_{i}}\right)}^{4}+\frac{3}{5}\right)\\ 0,z_{i}>\sigma_{R} \end{array},z_{i}\leq \sigma_{R}$$

in accordance to [13].

#### 2.2.2 Integration scheme

A detailed description of the integration scheme can be found in ([14], Eq. (18)) and ([13], Eq. (1)). A simplified notation of the algorithm is given by this formula

(4) $$\Delta r_{i}=\left[v_{sh\mathrm{ear}}\left(z_{i}\right){\;(\mu }_{\mathrm{ii}}/\mu_{0})\cdot \hat{x}+\sum_{j=1}^{N}\mu_{\mathrm{ij}}\cdot F_{\mathrm{ij}}+v_{\mathrm{corr}}\left(z_{i}\right)\cdot \hat{z}\right]\cdot \Delta t+{\Delta r}_{i}^{\mathrm{random}}\left(t\right),$$

where $v_{sh\mathrm{ear}}$ is the velocity of bead i due to the shear flow, $z_{i}$ is the third component of $r_{i}$, $\mu_{0}$ is a mobility given by Stoke’s formula, $\mu_{\mathrm{ij}}$ is the 3 dimensional submatrix of the Rotne–Prager–Blake tensor μ with $\mu_{3i-2,3j-2}$ being the upper left entry of that submatrix as defined in Eq. (5),

(5) $$\mu =\left(\begin{array}{ccc} \mu_{11} & \ldots & \mu_{1N}\\ \vdots & \ddots & \vdots \\ \mu_{N1} & \ldots & \mu_{\mathrm{NN}} \end{array}\right)=\left(\begin{array}{ccc} \left(\begin{array}{ccc} \mu_{1,1} & \ldots & \mu_{1,3}\\ \vdots & \ddots & \vdots \\ \mu_{3,1} & \ldots & \mu_{3,3} \end{array}\right) & \ldots & \left(\begin{array}{ccc} \mu_{1,N-2} & \ldots & \mu_{1,N}\\ \vdots & \ddots & \vdots \\ \mu_{3,N-2} & \ldots & \mu_{3,N} \end{array}\right)\\ \vdots & \ddots & \vdots \\ \left(\begin{array}{ccc} \mu_{N-2,1} & \ldots & \mu_{N-2,3}\\ \vdots & \ddots & \vdots \\ \mu_{N,1} & \ldots & \mu_{N,3} \end{array}\right) & \ldots & \left(\begin{array}{ccc} \mu_{N-2,N-2} & \ldots & \mu_{N-2,N}\\ \vdots & \ddots & \vdots \\ \mu_{N,N-2} & \ldots & \mu_{N,N} \end{array}\right) \end{array}\right),$$

$F_{\mathrm{ij}}$ is the force, that bead j exerts on the bead i and $\hat{x}$ and $\hat{z}$ are dimensionless unit vectors in x and z direction, respectively. The terms on the right hand side of Eq. (4) account for (in order left to right): the shear flow, all deterministic forces acting on bead i, a correction due to the spatial dependence of the random velocity, the random velocity (assumed to fulfill the fluctuation-dissipation theorem). Hydrodynamic interactions are treated through the mobility matrix and a detailed description of the employed mobility matrix can be found in [14] Eq. (7)ff.

#### 2.2.3 Parameter values

In our coarse-grained bead spring model (Eq. 2) each bead corresponds to a protomer of VWF with a reported radius of gyration of *a*=30 nm [9]. The cohesion strength was chosen to be 2$k_{B}T$, since this value leads to a collapsed conformation of the VWF multimer in the untethered case [10] and thus resembles the physiological situation of VWF in the bloodstream The spring constant for the interaction between two adjacent beads was set to $\kappa =\frac{20}{3}\frac{k_{B}T}{\mathrm{nm}}$. The first bead was put at the no slip boundary and fixed throughout the course of the simulation. We added a weak repulsive potential with a short range of $\sigma_{R}$=45 nm to make sure beads could not penetrate the wall. All other bead’s positions were updated using a time step of Δt=55 ns over the course of $10^{8}$ steps covering a time frame of 5.5 s. The time step Δt was calculated assuming T to be room temperature and the dynamic viscosity of the surrounding fluid to be 0.89 mPa s. Prior to the simulation run, an equilibration run of at least $10^{5}$ steps has been performed.

#### 2.2.4 Tensile force calculation

Block averages of the distance of adjacent beads $r_{i,i+1}$ were recorded over 100 steps. Those were again averaged at the end of the simulation run, yielding a mean distance $\langle r_{i,i+1}\rangle$ of adjacent beads. Mean tensile forces $f_{i}$ where then calculated from the equation

(6) $$f_{i}=\kappa \left(\left\langle r_{i,i+1}\right\rangle -2a\right).$$

### 2.3 Fitting of rupture forces versus loading rate profiles in the MD loading-rate range

Rupture forces were fitted in a broad loading-rate range covering both AFM and MD regimes, using the model recently developed by Bullerjahn, Sturm, and Kroy (BSK) [11] (Fig. 4(C) and (D), [1]). In this model, three parameters were used for the fitting: the energy barrier to overcome during rupture (*E*), the distance from the bound to the transition rupture state (*x*_b_), and the diffusion constant of the system along the pulling reaction coordinate *D*. For a given loading rate LR and parameters (*E*, *xb*, *D*), the BSK method yields the probability distribution of rupture forces: *P*(*F*; *LR*, E, *xb*, *D*). Accordingly, a maximum likelihood approach was used in conjunction to the BSK model to determine the set of parameters that best described the entire set of rupture forces recovered at different applied loading rates. Fitting was carried out considering, the AFM data of the VWF A1-A2 complex either in its wild-type (A1/A2) or its bridged (A1/[A2]) form (Fig. 5). AFM data used for fitting was obtained as explained as follows. Histograms of the AFM rupture forces were computed for each pulling velocity. Peaks in the histograms were extracted and a Gaussian distribution was fitted around the peak with the lowest force. This peak was considered for the analysis because it corresponds in most of the cases to the fingerprint of the VWF A1-A2 wild-type rupture process (see Fig. 1(B)). Resulting Gaussian distributions were considered as the input for the maximum likelihood BSK-based approach. The loading rate (LR) was estimated as the product LR=V<ke>, where V is the pulling velocity and ke is the effective spring constant of the linkers, cantilever, and VWF proteins. ke was determined from the slope of each force distance cycle. <> denotes average over cycles. Note that the elastic constant ks of the pulling apparatus (cantilever and linkers) should be used instead. Nevertheless, if the protein is sufficiently stiff and thereby its elastic constant is substantially large (kp→∞), then the effective elastic constant corresponds to that of the pulling apparatus (1/ke=1/ks+1/kp≈1/ks). Indeed, kp was found to almost three orders of magnitude larger than ke, thus justifying our use of ke as an estimate of ks. Fitting was also performed by combining AFM data with MD data, for both A1-A2 wild-type and bridged construct variants. MD rupture forces recovered from the smoothed force-distance profiles were used (Fig. 4, [1]). For the wild-type construct, all MD rupture forces were considered. For the bridged construct, the largest force value was excluded as it was obtained from a simulation involving the unfolding of A2, a situation that is prevented for this construct. The pulling velocity in the force-probe MD simulations was *V*=0.2 m/s and the spring constant of the two virtual springs (connected in series) was 415.145 pN/nm. Therefore the MD loading-rate was found to be *LR*=8.3×1010 pN/s. The activation rate k0 in the absence of force was estimated as a function of the three fitting parameters based on Kramers rate theory as [12]:

$$k_{0}\frac{2\mathrm{DE}}{x_{b}^{2}k_{B}T}\sqrt{\frac{E}{\pi k_{B}T}}{\;e}^{-E/k_{B}T},$$

where *k_B_* is the Boltzmann temperature and *T* the temperature.
